# Supplementary material for: Targeted Antisense Oligonucleotide-Mediated Skipping of Murine Postn Exon 17 Partially Addresses Fibrosis in D2.mdx Mice
Source: Int J Mol Sci. 2024 Jun 1;25(11):6113. doi: 10.3390/ijms25116113 (PMC11172600; doi:10.3390/ijms25116113)
Supplement: Supplementary file 1 [file ijms-25-06113-s001.zip › ijms-3008413-supplementary.pdf]

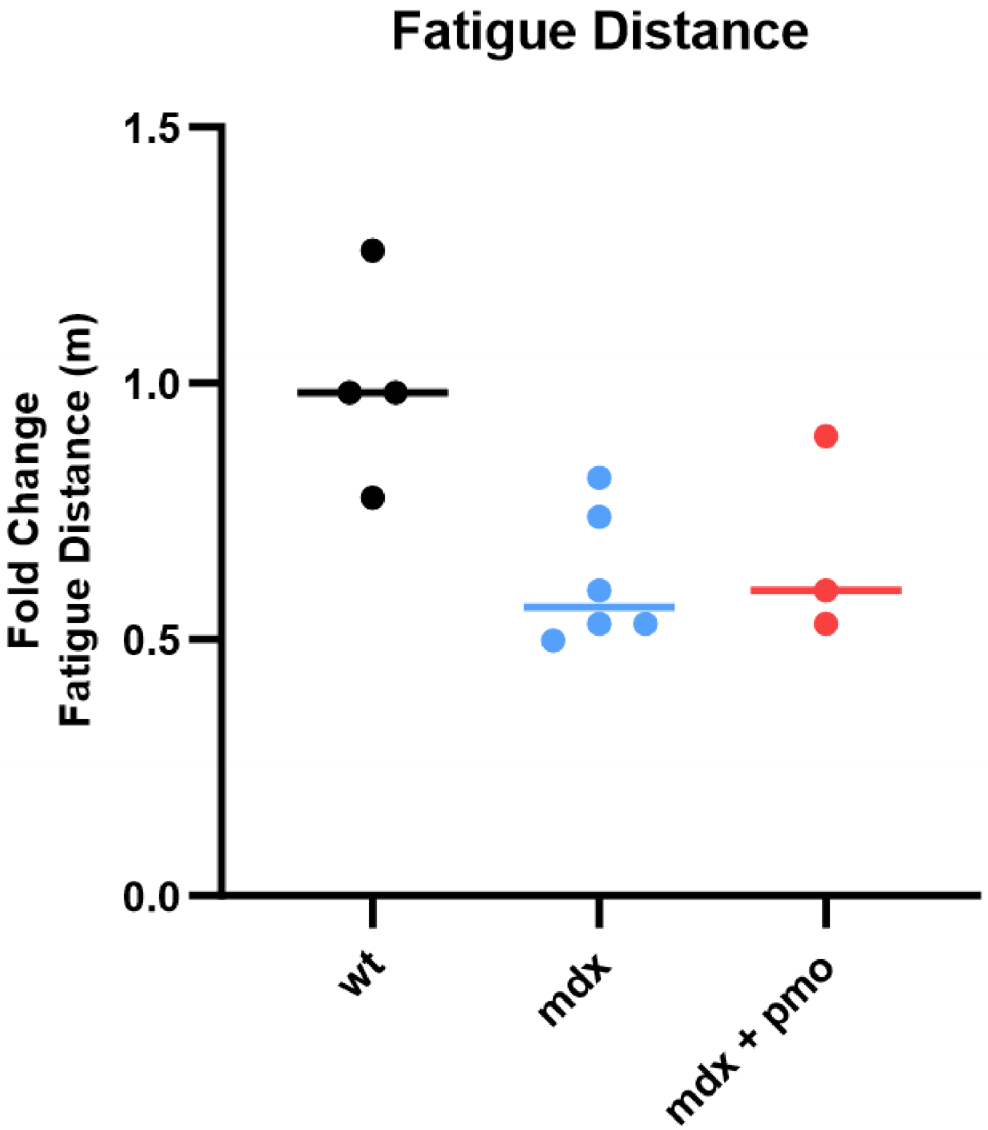

Supplementary Figure S1. vivoPMO mediated mPostn exon 17 skipping has no significant effect on fatigue distance. Fatigue distance was measured using the Treadmill Simplex II apparatus (Columbus Instrumentation, Columbus, Ohio, USA) featuring a 15% incline. A period of 5 minutes was allotted for the mice to acclimatise to the equipment before the commencement of the assessment. Subsequently, the treadmill was initiated at an initial velocity of 5 m/min for the initial 5 minutes, following which the velocity was augmented by 0.5 m every 1 minute. The determination of exhaustion in the animals was predicated upon their inability to evade a stopper placed on the treadmill for a duration of 10 seconds. Distance was calculated using the time to fatigue recorded and represented as fold change over wt. Outliers were identified via the ROUT method and omitted from statistical analysis. Data analysed by 1 way ANOVA and no significance was recorded ($p>0.05$). Error bars = +/- SD, qPCR in triplicate.
